# Prevalence, Risk Factors, and Complications of Sickle Cell Disease in Saudi Arabia: A Systematic Review

**DOI:** 10.7759/cureus.65263

**Published:** 2024-07-24

**Authors:** Nazim F Hamed, Yousef Dakheel Allah Alatawi, Danya Mohammed Zuhair AlKabbani

**Affiliations:** 1 General Pediatrics, Maternity and Children Hospital, Tabuk, SAU; 2 Pediatric Hematology and Oncology, Security Forces Hospital Dammam, Dammam, SAU; 3 Hematology, Security Forces Hospital Dammam, Dammam, SAU

**Keywords:** hemoglobinopathies, systematic review, complications, risk factors, prevalence, saudi arabia, sickle cell disease

## Abstract

This study examined sickle cell disease (SCD) in Saudi Arabia. A systematic search of relevant databases was conducted to identify studies investigating SCD in the Saudi population. Studies were then screened based on predefined criteria and critically appraised for methodological quality. Data was extracted and synthesized to provide an overall picture of the SCD burden in Saudi Arabia. The most commonly reported complications were vaso-occlusive crises (VOC), acute chest syndrome (ACS), acute painful crisis, splenic sequestration, osteomyelitis, aplastic crisis, hemolytic crisis, serious bacterial infections, chronic vascular occlusion (CVO), depression, sickle cell nephropathy (SCN), obstructive sleep apnea (OSA), and renal complications. Reduced blood levels of antioxidant trace elements (Cu, Zn, and Se) may encourage oxidative stress, which in turn may contribute to the pathophysiology of SCD. Infections and ACS were common among young children (<7 years) while pain attacks were common in older children (>7 years). The high rate of hospitalizations among SCD patients highlights the need for better management strategies. Future research should focus on understanding the underlying causes of SCD complications and developing new ways to control them.

## Introduction and background

Sickle cell disease (SCD) is a genetic disorder caused by a mutation in the gene encoding beta-globin, a subunit of hemoglobin, the oxygen-carrying protein in red blood cells. In individuals with SCD, hemoglobin is abnormal, causing red blood cells to become sickle-shaped and less flexible. This can lead to a variety of complications, including pain crises, organ damage, and even death. SCD is a major public health concern, particularly in countries with a high prevalence of the disease, such as Saudi Arabia [1].

The prevalence of SCD in Saudi Arabia is estimated to be around 2-4% of the population, with higher rates reported in some regions. This makes it one of the most common genetic disorders in the country. The high prevalence of SCD in Saudi Arabia can be attributed to a combination of factors, including a high rate of consanguineous marriages, where individuals marry within their own families, leading to an increased risk of inherited genetic disorders [2].

One of the key risk factors for SCD in Saudi Arabia is a family history of the disease. Individuals who have a family member with SCD are at an increased risk of being carriers of the gene mutation that causes the disease. Consanguineous marriages also play a significant role in the transmission of the SCD gene, as the likelihood of both parents being carriers of the gene is higher when they are closely related. Other risk factors for SCD in Saudi Arabia include a lack of awareness about the disease, inadequate access to genetic counseling and testing, and limited availability of treatment options [3].

Complications of SCD can vary widely, depending on the severity of the disease and the individual's overall health. One of the most common complications of SCD is a vaso-occlusive crisis, also known as a pain crisis. This occurs when sickle-shaped red blood cells block blood vessels, leading to severe pain and tissue damage. Other complications of SCD can include anemia, infections, acute chest syndrome, stroke, and organ damage. These complications can significantly impact the quality of life of individuals with SCD and may require ongoing medical treatment and management [4,5].

In Saudi Arabia, efforts have been made to raise awareness about SCD and provide support for individuals and families affected by the disease. The Saudi Ministry of Health has implemented programs to increase access to genetic counseling and testing, as well as to promote early detection and treatment of SCD. Additionally, specialized treatment centers have been established in major cities to provide comprehensive care for individuals with SCD [6].

SCD is a significant public health issue in Saudi Arabia, with a high prevalence and a range of risk factors and complications. Efforts to raise awareness, improve access to genetic counseling and testing, and provide specialized care are essential to address the challenges posed by SCD in the country. By increasing knowledge and support for individuals with SCD, Saudi Arabia can improve outcomes and quality of life for those affected by this genetic disorder [3].

SCD is a genetic blood disorder that affects a significant portion of the population in Saudi Arabia. Understanding the prevalence, risk factors, and complications of SCD in the Saudi Arabian population is crucial for effective management and prevention strategies. Despite the high prevalence of SCD in Saudi Arabia, there is a lack of comprehensive data on the specific risk factors and complications associated with the disease in this population. This knowledge gap hinders the development of targeted interventions and optimal healthcare services for individuals affected by SCD in the country. This study aimed to conduct a systematic review to determine the prevalence, identify risk factors, and explore complications of SCD in Saudi Arabia. By addressing these key aspects, this study aimed to provide a comprehensive understanding of the disease landscape in the country.

The study aimed to compile and analyze current research on the frequency of sickle cell disease (SCD) in Saudi Arabia. It seeks to examine the risk factors linked to the onset of SCD in the Saudi Arabian populace, as well as delve into the prevalent complications and health results among those affected by SCD in the region. Additionally, the research evaluated how socioeconomic elements influence the treatment and consequences of SCD in the Saudi Arabian setting.

## Review

Methods

For this systematic review, we followed the recommendations outlined in the Preferred Reporting Items for Systematic Reviews and Meta-Analyses (PRISMA) [7]. An electronic search was performed on databases such as PubMed, Web of Science, Scopus, and ScienceDirect. The search terms used will be specific to SCD, risk factors, prevalence, and complications. Relevant keywords were included in the search strategy for these situations. Independently, two reviewers went through the search results, chose pertinent papers, collected data, and used the right assessment methods to determine how good the included research was.

Inclusion and Exclusion Criteria

Studies conducted in Saudi Arabia; studies published in the English language; studies that focus on the prevalence, risk factors, or complications of SCD; studies that include information on the Saudi Arabian population; primary research studies, including cross-sectional studies, cohort studies, case-control studies, and clinical trials; studies published within the last 10 years to ensure relevance were included in this review.

Studies not conducted in Saudi Arabia; studies published in languages other than English; studies focusing on other types of hemoglobinopathies or blood disorders unrelated to SCD; review articles, case reports, editorials, and letters to the editor; studies with inadequate data or insufficient information on SCD prevalence, risk factors, and complications; studies with a high risk of bias or poor methodological quality; studies published more than 10 years ago were excluded for this review.

Data Extraction

Rayyan (Doha, Qatar: Qatar Computing Research Institute {QCRI}) was used to validate the search results in order to guarantee accuracy [8]. The inclusion and exclusion criteria were used to determine the relevancy of the titles and abstracts that the search produced. Papers that satisfied the inclusion requirements were carefully examined by the study team. Consensus was used to settle disagreements. Using a predetermined data extraction form, key study data, such as titles, authors, publication year, study location, gender distribution, participant demographics, age at first SCD diagnosis, prevalence of SCD, complications, and risk factors, were documented. An impartial assessment instrument was created to evaluate the possibility of bias.

Data Synthesis Strategy

Summaries of the research findings and elements were created utilizing information taken from pertinent studies in order to offer a qualitative assessment. The best method for making use of the data from the studies that were included was decided upon after the data collection for the systematic review was finished.

Risk of Bias Assessment

The Joanna Briggs Institute (JBI) critical assessment criteria for studies reporting prevalence data were utilized to assess the study's quality [9]. This tool had nine questions. A score of one was given for a positive response, while a score of zero was given for a negative, ambiguous, or irrelevant response. The scores were categorized as low quality for scores below 4, moderate quality for scores between 5 and 7, and high quality for scores above 8. The quality of the studies was evaluated by researchers independently, and differences were settled through discussion.

Results

Systematic Search Outcomes

**Figure 1:**
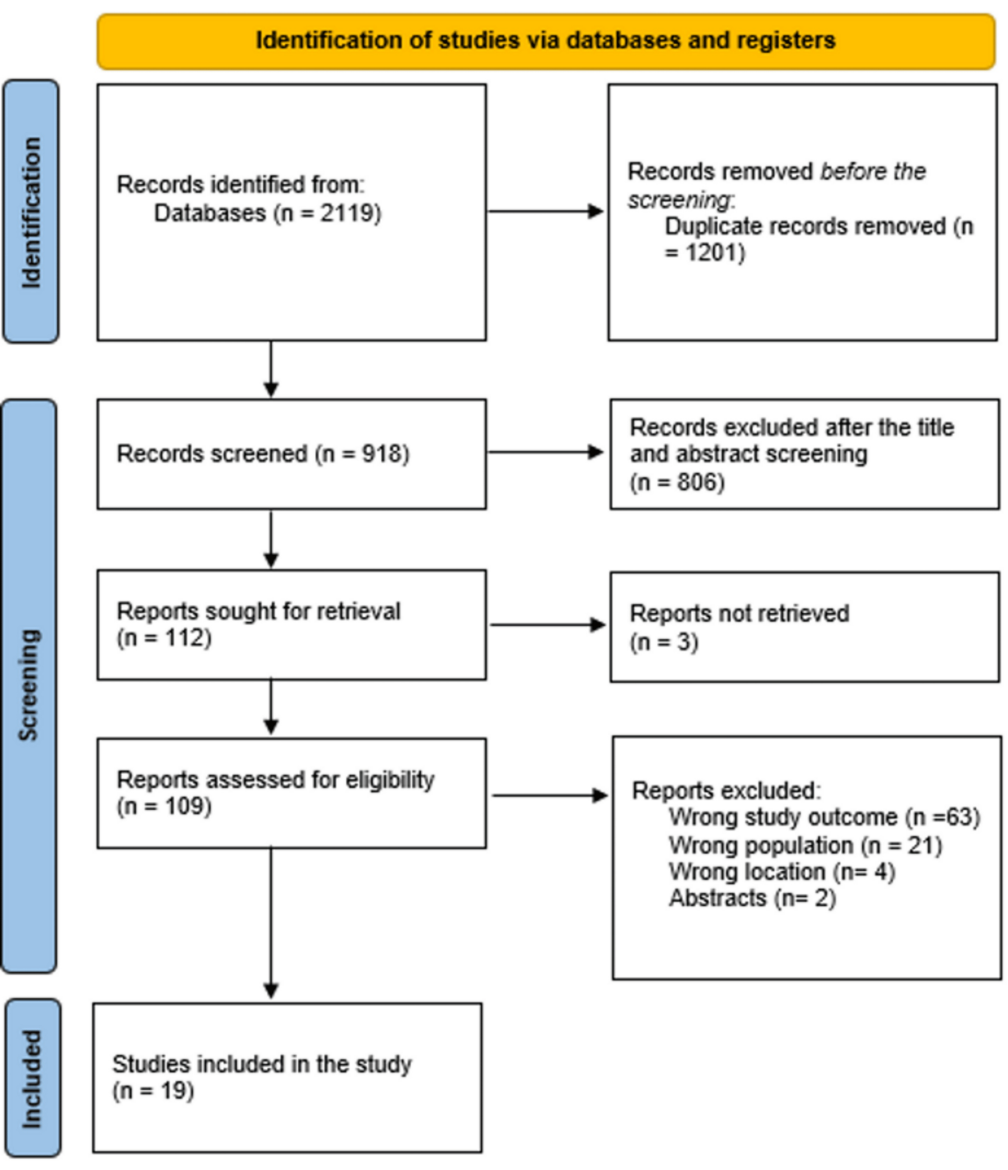
Study decision is summed up in a PRISMA diagram. PRISMA: Preferred Reporting Items for Systematic Reviews and Meta-Analyses

After 1201 duplicates were removed, a total of 2119 study papers were found through a systematic search. After 918 studies had their titles and abstracts evaluated, 806 papers were discarded. Merely three articles were not located out of the 112 reports that were required to be retrieved. A total of 109 articles passed the screening process for full-text evaluation; among the excluded articles, 63 were rejected due to incorrect study results, 21 due to incorrect population type, four articles for wrong location, and two were abstracts. Nineteen research publications in this systematic review satisfied the eligibility requirements. An overview of the procedure used to choose the research is illustrated in Figure 1.

Sociodemographic Features of the Comprised Studies

The research publications' sociodemographic information is displayed in Table 1. Nineteen studies, including a total of 6649 participants, of whom 3630 (54.6%) were males, were included in our data [10-28]. Eleven studies were retrospective cohorts [10,12-14,17-19,21,25-27], seven were cross-sectional [11,17,20,22-24,28], and one was case-control [14]. Seven studies were conducted in Jeddah [12,15,19,22,23,26,27], five in Riyadh [11,17,18,20,24], two in Jazan [16,28], two in Al-Khobar [10,21], one in Al Madinah [13], one in Tabuk [14], and one in Al Ahsa [25]. The earliest studies were conducted in 2015 [15,17] and the latest in 2024 [19].

**Table 1:** The sociodemographic attributes of the participating populations.

Study	Study design	City	Participants	Mean age	Males (%)
Yousef et al. (2022) [10]	Retrospective cohort	Al‑Khobar	42	7.18±3.38	22 (52.4%)
Hasanato (2019) [11]	Cross-sectional	Riyadh	33	8.5±4.1	19 (57.5%)
Abd El-Ghany et al. (2021) [12]	Retrospective cohort	Jeddah	94	7.29±3.82	56 (59.6%)
Abd Elmoneim et al. (2019) [13]	Retrospective cohort	Al Madinah	739	14.2±2.1	414 (56%)
Hanafy et al. (2020) [14]	Retrospective cohort	Tabuk	58	9	33 (56.9%)
Sehlo and Kamfar (2015) [15]	Case-control	Jeddah	120	11.9	64 (53.3%)
AlAmeer et al. (2021) [16]	Cross-sectional	Jazan	484	1-15	237 (49%)
Elsayid et al. (2015) [17]	Retrospective cohort	Riyadh	3332	NM	1889 (56.7%)
Alzomor et al. (2022) [18]	Retrospective cohort	Riyadh	30	3.7±2.7	17 (57%)
Basuni et al. (2023) [19]	Retrospective cohort	Jeddah	101	NM	58 (57.4%)
Alotaibi et al. (2018) [20]	Cross-sectional	Riyadh	70	6.5-11	39 (55.7%)
Yousef et al. (2022) [21]	Retrospective cohort	Al-Khobar	42	6-24	22 (52.4%)
Alzahrani et al. (2020) [22]	Cross-sectional	Jeddah	322	2-18	165 (51.2%)
Abulhamail et al. (2022) [23]	Cross-sectional	Jeddah	150	9.5±4.3	78 (53%)
Al-Otaibi et al. (2017) [24]	Cross-sectional	Riyadh	65	8.1	32 (49.2%)
Alsaif et al. (2021) [25]	Retrospective cohort	Al Ahsa	320	5.3±2.9	185 (58%)
Basuni et al. (2023) [19]	Retrospective cohort	Jeddah	99	NM	49 (49.4%
Alzahrani et al. (2021) [27]	Retrospective cohort	Jeddah	102	7.9±4.2	45 (44%)
Hazzazi et al. (2020) [28]	Cross-sectional	Jazan	446	27.9±23.6	206 (51.2%)

Clinical Outcomes

The clinical features are displayed in Table 2. Only three studies reported the participants' age at SCD diagnosis [10,19,26]. Moreover, only two articles reported the prevalence of SCD in 9.2% and 22.6% of the studied Saudi population [17,28]. The most commonly reported complications were vaso-occlusive crises (VOC), acute chest syndrome (ACS), acute painful crisis, splenic sequestration, osteomyelitis, aplastic crisis, hemolytic crisis, serious bacterial infections, chronic vascular occlusion (CVO), depression, sickle cell nephropathy (SCN), obstructive sleep apnea (OSA), and renal complications. Reduced blood levels of antioxidant trace elements (Cu, Zn, and Se) may encourage oxidative stress, which in turn may contribute to the pathophysiology of SCD [11]. Infections and ACS were common among young children (<7 years), while pain attacks were common in older children (>7 years) [12,13]. One study reported that the male gender is a potential risk factor for developing SCD [17]. A history of periodic limb movements and adenotonsillar hypertrophy were two of the numerous variables linked to OSA in SCD patients [23].

**Table 2:** Clinical features and results of the included research. NM: not-mentioned; VOC: vaso-occlusive disorder; AVN: avascular necrosis; ACS: acute chest syndrome; CVA: cerebrovascular accident; PRES: posterior reversible encephalopathy syndrome; TIA: transient ischemic attack; SAH: subarachnoid hemorrhage; SCN: sickle cell nephropathy; SBI: serious bacterial infections; UTI: urinary tract infection; OSA: obstructive sleep apnea; PHTN: pulmonary hypertension; SCD: sickle cell disease; HbSS: hemoglobin sickle cell disease; OSAS: obstructive sleep apnea syndrome

Study	Age at first diagnosis	Prevalence of SCD (%)	Complications	Risk factors	JBI
Yousef et al. (2022) [10]	6.6±3.4	NM	VOC (76.2%), splenic sequestration (19%), osteomyelitis (19%), aplastic crisis (14.3%), hemolytic crisis (11.9%), thromboembolic events (7.1%), cholecystitis (2.4%), AVN (2.4%), sepsis (2.4%)	NM	Moderate
Hasanato (2019) [11]	NM	NM	Painful episodes/year (4.3±2.7) and infections/year (5.1±1.7)	Reduced blood levels of antioxidant trace elements (Cu, Zn, and Se) may encourage oxidative stress, which in turn may contribute to the pathophysiology of SCD	High
Abd El-Ghany et al. (2021) [12]	NM	NM	VOC (64.9%), pain in the limbs (45.7%), infections (24.5%), ACS (18.1%), acute hemolytic crisis (12.8%)	NM	Moderate
Abd Elmoneim et al. (2019) [13]	NM	NM	Acute painful crisis (49.7%), ACS (20.9%), infections (17.5%), acute anemia (8.1%), hand-foot syndrome (2%), stroke (1.3%), priapism (0.5%)	NM	Moderate
Hanafy et al. (2020) [14]	NM	NM	ACS (30.1%), CVA (18.2%), other neurologic (PRES, TIA, SAH, acute soft head) (5.37%), GI emergency (pancreatitis, liver failure, hepatic sequestration) (4.3%), splenic sequestration (2.15), sepsis (1.07%)	NM	Moderate
Sehlo and Kamfar (2015) [15]	NM	NM	Depression (13%)	NM	Moderate
AlAmeer et al. (2021) [16]	NM	NM	SCN (24.8%), ACS (32.4%), hypersplenism (21.3%), ischemic stroke (4.8%)	HbSS was considerably higher in SCN patients (p=0.027)	Moderate
Elsayid et al. (2015) [17]	NM	307 (9.2%)	NM	Out of all SCD patients, males are more likely than females to have the disease (56.4%). The incidence of SCD was higher in children (48.5%)	High
Alzomor et al. (2022) [18]	NM	NM	UTI (2.2%), bacteremia (1.3%), osteomyelitis (0.24%), meningitis (0.12%)	NM	Moderate
Basuni et al. (2023) [19]	23±20 (months)	NM	VOC (65%), ACS (46%), splenic sequestration (31%), hyperhemolysis (23%), aplastic crisis (4%)	NM	Moderate
Alotaibi et al. (2018) [20]	NM	NM	OSA (46%)	NM	Moderate
Yousef et al. (2022) [21]	NM	NM	ACS (59.5%)	Recurrence was substantially correlated with younger age at first ACS (p=0.003), higher mean values of baseline reticulocyte (p=0.036), mean corpuscular volume (MCV) (p=0.011), and baseline white blood count (WBC) (p=0.009)	Moderate
Alzahrani et al. (2020) [22]	NM	NM	Pneumonia (9.3%), ACS (9.6%), spleen sequestration (7.5%), osteomyelitis (7.1%), stroke (4.3%), gallbladder stones (3.4%), dactylitis (0.9%), priapism (0.9%), AVN (0.9%), aplasia (0.6%)	NM	Moderate
Abulhamail et al. (2022) [23]	NM	NM	OSA (22%)	After correction, children with periodic limb movements had nine times higher odds of having OSAS than children without such movements. A history of periodic limb movements and adenotonsillar hypertrophy were two of the numerous variables linked to OSAS	Moderate
Al-Otaibi et al. (2017) [24]	NM	NM	OSA (80%)	NM	Moderate
Alsaif et al. (2021) [25]	NM	NM	Overall SBI prevalence (8%): pneumonia (3%), osteomyelitis (2.50), bacteremia (0.9%), UTI (0.9%)	NM	High
Basuni et al. (2023) [19]	18 (months)	NM	Hematuria (38%), proteinuria (11%), hyposthenuria (4%), glomerular hyperfiltration (7%), glomerular dysfunction (1%)	NM	Moderate
Alzahrani et al. (2021) [27]	NM	NM	VOC (32%), UTI (38%), osteomyelitis (23%), meningitis (7%), ischemic stroke (16%), AVN (13%), gallstones (4%), PHTN (1%)	There was a strong correlation (p<0.05) between the development of complications and patients' raised systolic blood pressure (SBP), hypoxia, and high white blood cell (WBC) counts. These patients also experienced greater complications	Moderate
Hazzazi et al. (2020) [28]	NM	91 (22.6%)	VOC (58.2), ACS (14.3), gallstone/cholecystitis (6.6), splenomegaly (5.5), osteomyelitis (4.4), AVN of the hip (1.1), others (9.9)	NM	Moderate

Discussion

This systematic review aimed to investigate the prevalence, complications, and risk factors of SCD in Saudi Arabia within the literature published in the last 10 years. However, we found that only two articles reported the prevalence of SCD in 9.2% and 22.6% of the studied Saudi population [17,28].

Genetic illnesses such as SCD are prevalent in Saudi Arabia [29,30]. Regretfully, even though SCD is becoming less common across the board in Saudi Arabia, its prevalence is still higher than in other nations. Furthermore, the fact that SCD is widespread in southern and eastern Saudi Arabia suggests that the country's present efforts to combat this illness are still insufficient. The eastern province has a 145 cases/10,000 population prevalence, which is significantly higher than the incidence rates in the southern region (24 cases/10,000 population), the western area (12 cases/10,000 population), and the center of the province (six cases/10,000 population) [31]. The first documented case of SCD was identified in the 1960s [29,32]. Saudi Arabia has many obstacles in its fight against SCD. The frequency of consanguineous marriages (57.7%), which might reach >80% in some rural areas, is a significant obstacle [33]. Through awareness campaigns, the Saudi government is attempting to inform the public about the consequences of consanguineous marriages because these unions raise the chance of genetic illnesses. The government's Premarital Screening Program and Genetic Counseling Program are the two most effective initiatives to date. All couples must participate in the Premarital Screening Program, which is offered at no cost at all government facilities. This initiative seems to be working; in the six years that it has been in place, the number of voluntary cancellations of marriage offered by individuals who are at-risk has grown fivefold [30].

Genetic factors play a key role in the development of SCD, as the disease is inherited in an autosomal recessive pattern. This means that both parents must carry a copy of the gene mutation for their child to develop the disease. In Saudi Arabia, consanguineous marriages are common, which can increase the likelihood of individuals carrying the gene mutation coming together and passing it on to their offspring. This accounts for the high prevalence of SCD in the country [2]. Environmental factors also play a role in the development of SCD. The hot and arid climate of Saudi Arabia can lead to dehydration, which can trigger sickling episodes in individuals with the disease. Additionally, the lack of awareness and education about SCD among the population can lead to delayed diagnosis and treatment, increasing the risk of complications [15]. Socioeconomic factors can also impact the incidence of SCD in Saudi Arabia. Access to healthcare services, including genetic counseling and early screening, can be limited in certain parts of the country. This can result in a lack of awareness about the disease and its risk factors, leading to a higher prevalence of SCD among the population. [22] The pathophysiology of SCD is complex and involves a number of mechanisms that contribute to the clinical manifestations of the disease. One of the key features of SCD is the polymerization of hemoglobin S, which causes red blood cells to become stiff and sticky. This can lead to vaso-occlusive episodes, where the sickled cells block blood flow to various organs and tissues, causing pain and tissue damage [27,30]. In addition to vaso-occlusion, SCD can also lead to hemolysis or the breakdown of red blood cells. This can result in anemia, jaundice, and an increased risk of infections. The chronic inflammation and oxidative stress associated with SCD can also contribute to the development of complications such as acute chest syndrome and stroke [14].

We found that the most commonly reported complications were VOC, ACS, acute painful crisis, splenic sequestration, osteomyelitis, aplastic crisis, hemolytic crisis, serious bacterial infections, CVO, depression, SCN, OSA, and renal complications. Many investigations have documented possible clinical symptoms of SCD, even though most carriers never have problems and the carrier status is usually asymptomatic [34]. Research to produce high-quality data that scientifically clarify the health outcomes associated with SCD has been called for by the Sickle Cell Disease Association of America, the American Society of Hematology, and the Secretary's Advisory Committee on Heritable Disorders in Newborns and Children of the Department of Health and Human Services [35-37]. Several nations, including the United States, have mandated newborn screening for sickle hemoglobin, so families, patients, and healthcare professionals are also looking for high-quality information regarding clinical risk [38].

The prevalent infections and ACS in SCD patients may be explained by the fact that there are several reasons why patients with SCD are more vulnerable to infection. These include immunological inadequacies brought on by malnourishment, opsonization problems of encapsulated organisms, and splenic dysfunction. In fact, impairments in humoral, cellular, and innate immune function persist throughout life when splenic dysfunction and malnutrition occur at a young age [39].

While the Premarital Screening Program and free genetic consultation have shown to be highly successful in the Saudi population, further effective programs (such as newborn screening) must be implemented in Saudi Arabia to discover new cases of SCD. In other nations, for instance, parental counseling and follow-up care for impacted cases have decreased SCD-related morbidity and mortality [40]. Genetic counseling and screening, however, are hampered for Saudi patients by a number of factors, including restricted access to healthcare services, a shortage of qualified medical personnel, social and cultural stigmas, and religious convictions. Therefore, in order to assist the populace in overcoming these obstacles, Saudi Arabia needs to start awareness campaigns extremely early [41].

Additionally, there is a dearth of genetic research on SCD in Saudi Arabia. To assess the efficacy of population-based policies and interventions, interventional studies are required. The Premarital Screening Program's and free genetic counseling's achievements should encourage the Saudi government to concentrate on nationwide newborn screening, which could lessen the primary and secondary burdens of SCD. Additionally, keeping a current register would be helpful in determining the true burden of SCD [41].

## Conclusions

In conclusion, the findings suggest that Saudi sickle cell disease (SCD) patients experience a significant rate of hospitalization, often as a result of severe complications or chronic pain. The variable traits and types of SCD lead to multisystem involvement, playing a crucial role in the disease's pathophysiology. Moving forward, it is essential to conduct future prospective research to gain a deeper understanding of the underlying mechanisms of SCD and to develop effective management strategies aimed at controlling and mitigating these potentially life-threatening complications. This holistic approach to SCD management will not only improve patient outcomes but also enhance the overall quality of life for individuals affected by this disease in Saudi Arabia.

## References

[1] Azmet FR, Al-Kasim F, Alashram WM, Siddique K (2020). The role of hydroxyurea in decreasing the occurrence of vaso-occlusive crisis in pediatric patients with sickle cell disease at King Saud Medical City in Riyadh, Saudi Arabia. Saudi Med J.

[2] Sendy JS, Alsadun MS, Alamer SS, Alazzam SM, Alqurashi MM, Almudaibigh AH (2023). Frequency of painful crisis and other associated complications of sickle cell anemia among children. Cureus.

[3] Talha M, Osman B, Abdalla S, Mirghani H, Abdoon I (2022). Pediatric sickle cell disease in Sudan: complications and management. Anemia.

[4] Bin Zuair A, Aldossari S, Alhumaidi R, Alrabiah M, Alshabanat A (2023). The burden of sickle cell disease in Saudi Arabia: a single-institution large retrospective study. Int J Gen Med.

[5] Dairi M, Almatrfi SS, Alsharif MM (2022). Prevalence of psychological symptoms and its impact on the quality of life of sickle cell disease patients in Makkah, Saudi Arabia. Cureus.

[6] Almasoudi EA, Magliah SF, Alzwaihri AS, Aljuwaybiri AO, Alqahtani AS (2022). Incidence of eye complications among sickle cell disease patients in Jeddah, Saudi Arabia: a cross-sectional study. Ann Med Surg (Lond).

[7] Page MJ, McKenzie JE, Bossuyt PM (2021). The PRISMA 2020 statement: an updated guideline for reporting systematic reviews. Br Med J.

[8] Ouzzani M, Hammady H, Fedorowicz Z, Elmagarmid A (2016). Rayyan-a web and mobile app for systematic reviews. Syst Rev.

[9] Munn Z, Aromataris E, Tufanaru C (2019). The development of software to support multiple systematic review types: the Joanna Briggs Institute System for the Unified Management, Assessment and Review of Information (JBI SUMARI). Int J Evid Based Healthc.

[10] Yousef AA, Shash HA, Almajid AN (2022). Acute chest syndrome in pediatric sickle cell disease: a 19-year tertiary center experience. Annals of Thoracic Medicine.

[11] Hasanato R (2019). Alterations in serum levels of copper, zinc, and selenium among children with sickle cell anemia. Turk J Med Sci.

[12] Abd El-Ghany SM, Tabbakh AT, Nur KI, Abdelrahman RY, Etarji SM, Almuzaini BY (2021). Analysis of causes of hospitalization among children with sickle cell disease in a group of private hospitals in Jeddah, Saudi Arabia. J Blood Med.

[13] Abd Elmoneim AA, Al Hawsawi ZM, Mahmoud BZ, Bukhari AA, Almulla AA, Sonbol AM, Makhdoum AM (2019). Causes of hospitalization in sickle cell diseased children in western region of Saudi Arabia. A single center study. Saudi Med J.

[14] Hanafy E, Altoonisi M, Alatawi A (2020). Characteristics and outcomes of patients with sickle cell disease admitted to pediatric intensive care: a retrospective review. J Appl Hematol.

[15] Sehlo MG, Kamfar HZ (2015). Depression and quality of life in children with sickle cell disease: the effect of social support. BMC Psychiatry.

[16] AlAmeer MR, Alsarhan BK, Alsarhan LK (2021). Epidemiology of sickle cell nephropathy in sickle cell anemia children, Saudi Arabia. Med Sci.

[17] Elsayid M, Al-Shehri MJ, Alkulaibi YA, Alanazi A, Qureshi S (2015). Frequency distribution of sickle cell anemia, sickle cell trait and sickle/beta-thalassemia among anemic patients in Saudi Arabia. J Nat Sci Biol Med.

[18] Alzomor O, Aljobair F, Al Kasim F, Azmet F, Alorini S, Alshihayb Y, Bahamdan Y (2022). Frequency of serious bacterial infection among febrile sickle cell disease children in the era of the conjugate vaccine: a retrospective study. Int J Pediatr Adolesc Med.

[19] Basuni ZT, Monagel DA, Taha A, Ahmed N, Ahmed A (2023). Neurological abnormalities among pediatric patients with sickle cell disease in Saudi Arabia: a single-center retrospective study. Front Pediatr.

[20] Alotaibi W, Eltahir S, Rayis M (2018). Pediatric sickle cell disease and obstructive sleep apnea: a cross-sectional study in a tertiary pediatric center in Saudi Arabia. J Family Community Med.

[21] Yousef AA, Shash HA, Almajid AN (2022). Predictors of recurrent acute chest syndrome in pediatric sickle cell disease: a retrospective case-control study. Children (Basel).

[22] Alzahrani YA, Algarni MA, Alnashri MM (2020). Prevalence and risk factors for microalbuminuria in children with sickle cell disease at King Abdulaziz University Hospital: a retrospective cross-sectional study. Cureus.

[23] Abulhamail A, AlShebli A, Merdad L, Wali S, Jastaniah W, Abaalkhail B (2022). Prevalence of and risk factors for obstructive sleep apnea in children with sickle cell: a multicentric cross sectional study. Ann Hematol.

[24] Al-Otaibi T, Al-Qwaiee M, Faraidi H, Batniji F, Al-Otaibi F, Al-Harbi A (2017). Prevalence of obstructive sleep apnea in children with sickle cell disease at a tertiary hospital in Saudi Arabia. Saudi Med J.

[25] Alsaif MA, Abdulbaqi M, Al Noaim K, Aghbari M, Alabdulqader M, Robinson JL (2021). Prevalence of Serious Bacterial Infections in Children with Sickle Cell Disease at King Abdulaziz Hospital, Al Ahsa. Mediterr J Hematol Infect Dis.

[26] Monagel DA, Algahtani SS, Karawagh LA, Althubaity WD, Azab SA, Haneef DF, Elimam N (2023). Renal outcomes in pediatric patients with sickle cell disease: a single center experience in Saudi Arabia. Front Pediatr.

[27] Alzahrani F, Fallatah AM, Al-Haddad FM, Khayyat ST, AlMehmadi WM, AlQahtani BG, Alamri RS (2021). Risk factors and complications among pediatric patients with sickle cell anemia: a single tertiary center retrospective study. Cureus.

[28] Hazzazi AA, Ageeli MH, Alfaqih AM, Jaafari AA, Malhan HM, Bakkar MM (2020). Epidemiology and characteristics of sickle cell patients admitted to hospitals in Jazan region, Saudi Arabia. J Appl Hematol.

[29] Jastaniah W (2011). Epidemiology of sickle cell disease in Saudi Arabia. Ann Saudi Med.

[30] Memish ZA, Saeedi MY (2011). Six-year outcome of the national premarital screening and genetic counseling program for sickle cell disease and β-thalassemia in Saudi Arabia. Ann Saudi Med.

[31] Al-Qurashi MM, El-Mouzan MI, Al-Herbish AS, Al-Salloum AA, Al-Omar AA (2008). The prevalence of sickle cell disease in Saudi children and adolescents. A community-based survey. Saudi Med J.

[32] Al-Owain M, Al-Zaidan H, Al-Hassnan Z (2012). Map of autosomal recessive genetic disorders in Saudi Arabia: concepts and future directions. Am J Med Genet A.

[33] El-Hazmi MA, Al-Swailem AR, Warsy AS, Al-Swailem AM, Sulaimani R, Al-Meshari AA (1995). Consanguinity among the Saudi Arabian population. J Med Genet.

[34] Tsaras G, Owusu-Ansah A, Boateng FO, Amoateng-Adjepong Y (2009). Complications associated with sickle cell trait: a brief narrative review. Am J Med.

[35] Naik RP, Haywood C Jr (2015). Sickle cell trait diagnosis: clinical and social implications. Hematology Am Soc Hematol Educ Program.

[36] Jordan LB, Smith-Whitley K, Treadwell MJ, Telfair J, Grant AM, Ohene-Frempong K (2011). Screening U.S. college athletes for their sickle cell disease carrier status. Am J Prev Med.

[37] (2012). Statement on screening for sickle cell trait and athletic participation. https://www.hematology.org/advocacy/policy-news-statements-testimony-and-correspondence/policy-statements/2012/screening-sickle-cell-trait-athletic-participation#:~:text=ASH%20does%20not%20support%20testing,principles%20of%20public%20health%20ethics..

[38] Benson JM, Therrell BL Jr (2010). History and current status of newborn screening for hemoglobinopathies. Semin Perinatol.

[39] Ochocinski D, Dalal M, Black LV, Carr S, Lew J, Sullivan K, Kissoon N (2020). Life-threatening infectious complications in sickle cell disease: a concise narrative review. Front Pediatr.

[40] Wilson M, Forsyth P, Whiteside J (2010). Haemoglobinopathy and sickle cell disease. Contin Educ Anaesth Crit Care Pain.

[41] Alotaibi MM (2017). Sickle cell disease in Saudi Arabia: a challenge or not. J Epidemiol Glob Health.

